# Diffuse Large B-Cell Lymphoma: From Molecular Stratification to Precision Immunotherapy

**DOI:** 10.3390/cells15131188

**Published:** 2026-06-30

**Authors:** Akbar Pasha, Aayushi Velingkar, Ramita Sharma, Priyanka Tiwari, Manasi Mundada, Rohan Tewani, Dylan T. Jochum, Rashid Mir, Faiq Ahmed, Sugunakar Vuree, Gopal Gopisetty, Senthil J. Rajappa, Aisha Ahmad Al-Khinji, Mallick Saumyaranjan, Chengfeng Bi, Waseem G. Lone

**Affiliations:** 1 Indo-American Cancer Research Foundation, Basavatarakam Indo-American Cancer Hospital and Research Institute, Banjara Hills, Hyderabad 500034, Indiaramita.bioinfo11@gmail.com (R.S.);; 2 Department of Molecular Medicine, Jamia Hamdard, New Delhi 110062, India; priyankatiwari6219@gmail.com; 3 Department of Hemato-Oncopathology, Basavatarakam Indo-American Cancer Hospital and Research Institute, Banjara Hills, Hyderabad 500034, India; 4 Medical Oncology, Basavatarakam Indo-American Cancer Hospital and Research Institute, Banjara Hills, Hyderabad 500034, India; 5 Department of Pathology, Microbiology and Immunology, University of Nebraska Medical Center, Omaha, NE 68198, USA; 6 Prince Fahad Bin Sultan Chair for Biomedical Research, University of Tabuk, Tabuk 71491, Saudi Arabia; 7 Department of Genetics, Basic Medical Sciences, Qatar University, Doha 2713, Qatar; 8 Department of Pathology, All India Institute of Medical Sciences, New Delhi 110029, India; 9 Division of Oncology & Hematology, Department of Internal Medicine, University of Nebraska Medical Center, Omaha, NE 68198, USA

**Keywords:** diffuse large B-cell lymphoma, cell-of-origin classification, genomic profiling, tumor microenvironment, personalized therapy, circulating tumor DNA

## Abstract

Diffuse large B-cell lymphoma (DLBCL) is a biologically heterogeneous mature B-cell neoplasm whose classification, prognosis, and therapy have been reshaped by advances in genomic, transcriptomic, epigenomic, single-cell, and spatial profiling technologies. This review focuses on how these approaches have refined the molecular landscape of DLBCL, including recurrent chromosomal translocations, tumor-suppressor alterations, oncogenic signaling pathways, and tumor-microenvironment programs. Cell-of-origin (COO) frameworks remain clinically useful. However, contemporary models extend beyond conventional germinal center categories by incorporating probabilistic genetic subtypes, expression-defined high-risk states, and spatially resolved lymphoma-cell and immune-cell ecosystems. These high-resolution methods clarify intratumoral heterogeneity, identify biologically distinct subgroups, and inform prognosis and therapeutic selection. The review also summarizes how tumor-intrinsic biology and the tumor-microenvironment (TME) shape responses to frontline therapy, targeted agents, antibody-drug conjugates, bispecific antibodies, and CD19-directed CAR T-cell therapy. Particular emphasis is placed on product-specific evidence in relapsed/refractory disease, rational sequencing of immunotherapies, and emerging biomarkers such as circulating tumor DNA-based measurable residual disease (ctDNA-MRD). Together, these developments support a shift from COO-centric classification toward dynamic, biology-driven models that incorporate tumor-intrinsic and microenvironmental determinants to guide personalized therapy in DLBCL.

## 1. Introduction

**Figure 1:**
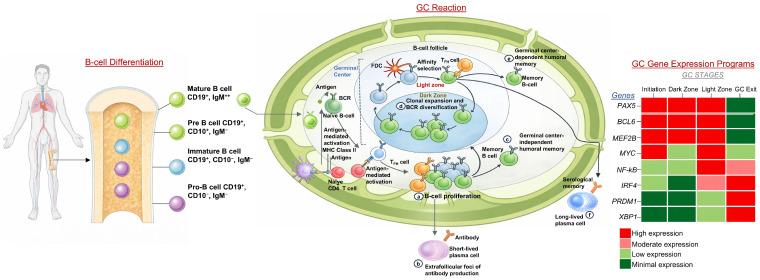
Germinal center dynamics and transcriptional programs driving B-cell differentiation and humoral memory formation: (**a**) a schematic illustration of B-cell development from bone marrow progenitors to mature peripheral B-cell populations. Immunophenotypes show developmental stages, from pro-B cells (CD19^+^, CD10^−^, IgM^−^) to immature B cells (CD19^+^, CD10^−^, IgM^−^), pre-B cells (CD19^+^, CD10^+^, IgM^−^), and mature naïve B cells (CD19^+^, IgM^++^). (**b**) Germinal center (GC) reaction in secondary lymphoid organs. Antigen-activated naïve B cells interact with T follicular helper (T_FH_) cells and enter the GC, where they cycle between the dark and light zones. The DZ is characterized by rapid clonal expansion and somatic hypermutation (SHM), whereas the LZ supports affinity-based selection via interactions with follicular dendritic cells (FDCs) and T_FH_ cells. (**c**) GC-dependent and GC-independent pathways of humoral memory formation. Following antigen stimulation, activated B cells differentiate through distinct developmental pathways to generate memory B cells and antibody-secreting plasma cells, thereby establishing durable humoral immunity and long-term immune protection. (**d**) Representative transcriptional programs associated with different GC stages. The heatmap depicts the relative expression levels of key regulatory genes during GC initiation, DZ, LZ, and GC exit. Color intensity represents relative gene expression (red—high expression, pink—moderate expression, light green—low expression, and dark green—minimal expression). (**e**) GC-dependent memory B-cell differentiation. Affinity-selected B cells emerging from iterative cycles of selection within the LZ differentiate into memory B cells that contribute to long-term humoral immunity. (**f**) Plasma-cell differentiation and the formation of serological memory. Activated B cells generate both short-lived plasma cells in extrafollicular responses and long-lived plasma cells that produce antibodies and provide durable serological immunity. Figure created using BioRender.com.

The germinal center (GC) reaction, a dynamic process occurring within secondary lymphoid organs, is characterized by class switch recombination (CSR) and the affinity maturation ofB cells [1,2]. Following the successful completion of V(D)J recombination and the generation of a functional B-cell receptor (BCR), naïveB cells migrate to secondary lymphoid organs. The GC begins through a complex process controlled by different transcription factors. This process is activated whenB cells encounter an antigen [2,3]. Within the GC’s dark zone (DZ), B cells rapidly proliferate and then undergo somatic hypermutation (SHM) of their immunoglobulin genes. Thus, this process diversifies the immunoglobulin variable regions of BCRs, generating a repertoire ofB cells with varying antigen-binding affinities [3,4]. These cells then move to the light zone (LZ), where they interact with follicular dendritic cells (FDCs), T follicular helper (T_FH_) cells, and macrophages. B-cell selection in the GC is based on how well they bind to antigens [3,5,6]. The cellular and molecular processes in the GC reaction are complex (Figure 1). B cells move repeatedly between the light and dark zones, undergoing several rounds of division and SHM, which facilitates affinity maturation.

At the transcriptional level, this cyclical process is tightly regulated. At the initiation of GC formation, *MYC* is transiently expressed. Following positive selection in the LZ, *MYC* expression is restored in LZB cells, which then migrate back to the DZ. In contrast, most cells in the DZ typically exhibit decreased *MYC* expression [3,7,8]. Within the GC reaction, *BCL6*, the main transcriptional regulator of the GC program, controls *MYC* activity, whereas the DZ phase has sustained BCL6 expression [3]. *BCL6* suppresses genes that are involved in DNA damage response and cell-cycle control [9,10]. Conversely, *EZH2* is predominantly expressed in matureB cells in the GC phase, where GC proliferation and maintenance depend on its activity. E2A, a transcription factor, is largely localized in the DZ of GC B cells. Cell cycle progression is driven by increased expression of *CCND3* and *E2F2*, along with reduced *RB1* activity. E2A also increases BCR signaling by blocking *PTPN6*, thereby activating SYK/BTK and downstream PI3K–AKT and NF-κB pathways. Additionally, *IRF4* shows a two-phase expression pattern in GC. It is first expressed in founder GCB cells, where it helps initiate the GC process, and is later re-expressed during plasma cell differentiation [7,10,11].

Early in GC formation, NF-κB is highly active. Its activity decreases in DZB cells and then increases again in LZB cells during selection. In contrast, BCL6 is highly active in GCB cells, especially in the DZ, and its activity decreases after cells exit the GC [3,10]. These genetic mechanisms that facilitate SHM and class switch recombination, which are essential for generating high-affinity immunoglobulin receptors of various types, also by increasing the susceptibility of B cells toward malignancy. Defects in these mechanisms lead to the emergence and progression of diverse B-cell lymphomas [12,13]. The aberrant activation of critical oncogenes and transcriptional regulators drives the malignant transformation of B cells. DLBCL is heterogeneous in origin: GCB-DLBCL derives from GC-B cells with a centrocyte-like LZ gene expression profile, whereas ABC-DLBCL arises from post-GC or activated B cells and is characterized by a block in differentiation at a plasmablast-like stage, associated with constitutive NF-κB signaling, and plasma cell differentiation (Figure 1) [3,12].

Recent reviews [14,15,16] have addressed advances in DLBCL genomics, TME biology, or novel therapeutic strategies, but these topics have largely been discussed in isolation. Herein, we aim to provide a comprehensive synthesis that contextualizes molecular classification systems, recurrent genetic alterations, single-cell and spatial transcriptomic findings, TME programs, and evolving immunotherapeutic strategies within a clinically relevant framework. This review discusses how molecular, cellular, and microenvironmental determinants collectively influence disease stratification, therapeutic selection, and precision immunotherapy in DLBCL. The review is based on seminal and recent studies retrieved from PubMed, Web of Science, and Scopus, using keywords related to DLBCL molecular classification, genomics, single-cell sequencing, spatial transcriptomics, TME, CAR T-cell therapy, bispecific antibodies, and precision immunotherapy. Landmark genomic studies, influential translational studies, and pivotal clinical trials that have substantially contributed to the biological understanding and clinical management of DLBCL were prioritized.

## 2. Beyond Cell-of-Origin: Molecular Determinants of DLBCL Heterogeneity and Evolution

**Figure 2:**
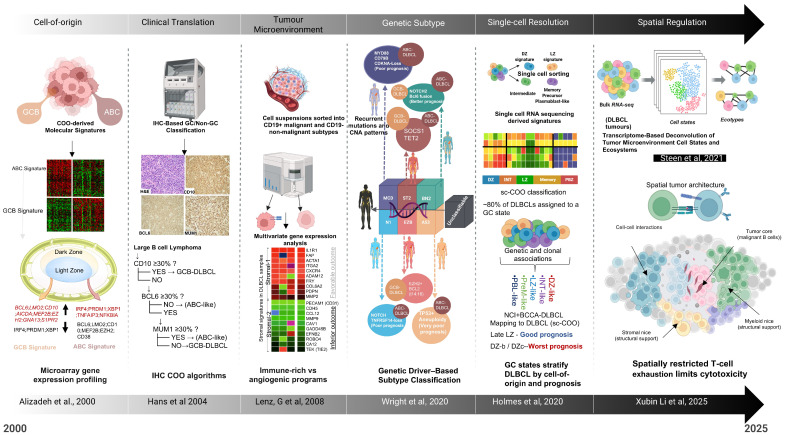
Evolution of DLBCL biology: from cell-of-origin to tumor ecosystem architecture. The first molecular classification by cell-of-origin (COO) using gene expression profiling (*Alizadeh et al., 2000*) [18] identified GCB and ABC subtypes. Translation to the clinic was achieved through immunohistochemistry-based algorithms (Hans et al., 2004) [19] and the identification of prognostically relevant TME programs (Lenz et al., 2008) [20]. Integration of genomic data defining genetic subtypes (Wright et al., 2020) [26], including MCD, BN2, EZB, N1, A53, and unclassified cases, refined the biological heterogeneity of DLBCL and revealed distinct pathogenic mechanisms, clinical outcomes, and therapeutic vulnerabilities. Advances in single-cell and spatial transcriptomics (X. Li et al., 2025; Holmes et al. (2020); Steen et al., 2021) [15,31,32] enabled high-resolution mapping of cellular states, ecosystem organization, and cell–cell interactions, highlighting the importance of spatial context and TME architecture in shaping disease biology and therapeutic response. Figure created using BioRender.com.

GEP and mutation analysis have greatly aided our understanding of the biology of DLBCL. In addition to COO, GEP analyses have identified additional prognostic transcriptional programs, including signatures enriched in cellular proliferation, stromal components of the TME, and major histocompatibility complex (MHC) class I and II [17,18]. Furthermore, the advent of molecular classification systems for DLBCL facilitated their application in immunohistochemistry, especially through the Hans algorithm. This algorithm stratifies tumors into germinal center B-cell (GCB) and non-GCB subtypes, predicated on the expression patterns of CD10, BCL6, and MUM1, as illustrated in Figure 2 [19]. This approach effectively mimics GEP-identified survival differences while maintaining prognostic importance independent of the International Prognostic Index (IPI) [19].

Notably, increased proliferative activity, alongside diminished MHC expression, correlates with unfavorable clinical outcomes; conversely, augmented stromal gene expression is associated with improved prognoses. Further investigation of the stromal signature has revealed two biologically distinct subcomponents: stromal-1, which reflects extracellular matrix deposition and immune-cell infiltration and is associated with a favorable outcome, and stromal-2, which is characterized by angiogenesis-related gene expression and is associated with a lower survival rate [20] (Figure 2). The considerable biological and clinical differences among groups classified by COO suggest that other genetic factors influence disease progression [21]. Accordingly, contemporary classification frameworks have incorporated these biological insights into standardized diagnostic systems.

Current disease classification should be framed using the fifth edition of the World Health Organization Classification of Haematolymphoid Tumors (WHO-HAEM5) and the International Consensus Classification (ICC) [22,23]. In addition to DLBCL, not otherwise specified (NOS), these classifications recognize several distinct large B-cell lymphoma entities, including EBV-positive DLBCL, NOS, T-cell/histiocyte-rich large B-cell lymphoma, intravascular large B-cell lymphoma, and fluid overload-associated large B-cell lymphoma [22,23]; however, the present review focuses primarily on DLBCL. In these systems, DLBCL, NOS, remains a diagnosis of exclusion within large B-cell lymphomas, and biological or therapeutic studies must be interpreted in distinction from primary mediastinal large B-cell lymphoma and from HGBCL with *MYC*-associated rearrangements. Importantly, WHO-HAEM5 and ICC both recognize that lymphomas with *MYC* and *BCL2* rearrangements represent a biologically coherent aggressive category, whereas *MYC/BCL6*-rearranged cases are more heterogeneous. Accordingly, in WHO-HAEM5, *MYC/BCL6*-rearranged tumors are no longer grouped with *MYC/BCL2*-rearranged “double-hit” lymphomas but are classified as DLBCL, NOS, or HGBCL, NOS; in contrast, ICC retains *MYC/BCL6*-rearranged disease as a provisional entity. This distinction is important because it underscores that formal diagnostic classification and biologic risk stratification are related but not identical frameworks [24].

Against this diagnostic background, several genomic classification systems have refined the biologic heterogeneity that remains unresolved by COO alone. Chapuy et al. integrated recurrent mutations, somatic copy-number alterations, and structural variants to identify reproducible molecular subtypes that differ prognostically beyond COO [21]. Similarly, Schmitz et al. linked recurrent combinations of driver lesions to distinct pathogenic mechanisms. They defined four major genetic subtypes: MCD, BN2, N1, and EZB, each with characteristic mutational patterns, signaling dependencies, microenvironmental features, and clinical outcomes [25]. LymphGen further extended this framework by probabilistically assigning DLBCL cases to genetically defined subtypes, including the additional ST2 and A53 subtypes, while also accommodating unclassified cases, thereby capturing clonal heterogeneity and evolutionary trajectories more effectively than binary COO classification alone [26]. Among the LymphGen-defined subtypes, A53 is characterized by frequent *TP53* alterations and aneuploidy and is consistently associated with inferior clinical outcomes. In contrast to other molecular subtypes, there are no well-defined targeted treatment approaches for A53 at present, representing an important unmet clinical need in DLBCL. The Chapuy and LymphGen classification systems show a high degree of overlap, despite being derived by distinct analytical approaches. Overall, the Chapuy C5 cluster corresponds to the *MYD88/CD79B*-driven MCD subtype, and C3 overlaps significantly with EZB tumors with *BCL2* alterations and germinal center B-cell features. However, the correspondence between these systems is not absolute, reflecting differences in methodology, genomic features incorporated, and cohort composition. Despite these important advances, the models do not fully capture the biology of the most aggressive germinal center-derived tumors. Studies of *MYC*-rearranged and HGBCLs show that aggressive disease exists along a biologic continuum that overlaps with, but is not fully defined by, conventional cytogenetic categories. Our prior integrative genomic analyses identified a subset of adult HGBCLs with a Burkitt-like gene expression program characterized by dysregulation of the *MYC*-ARF-*MDM2*-p53 axis and activation of B-cell receptor-driven PI3K and NF-κB signaling [27]. More recent analyses of HGBCL, not otherwise specified (NOS), similarly support marked biologic heterogeneity, with some tumors showing DLBCL-like transcriptional programs and complex genomic alterations affecting B-cell activation, epigenetic regulation, and cell-cycle control [28]. Together, these findings support the concept that high-grade biology cannot be inferred from morphology or FISH categories alone. This is particularly relevant within GCB-DLBCL, where high-risk biology is not fully captured by conventional fluorescence in situ hybridization. The double-hit gene-expression signature (DHITsig) and the molecular high-grade classifier identify aggressive DZ-like disease enriched for, but not limited to, *MYC/BCL2*-rearranged tumors, thereby refining risk stratification beyond cytogenetically defined double-hit lymphoma alone [29,30].

Recent advances in genomic and transcriptome profiling have highlighted the need for more biologically defined models of lymphoma cell of origin. In this context, single-cell transcriptomic analyses of human GCB cells show that GC biology goes beyond the traditional DZ and LZ frameworks, revealing a continuum of transcriptionally connected intermediate states as well as discrete precursors of memory B and plasma cells [31]. A single-cell-resolved COO (sc-COO) model assigns the majority of DLBCLs to defined stages of normal GC differentiation, enhancing and refining the conventional GCB and ABC classifications (Figure 2) [31]. These lymphoma subgroups, identified at the single-cell level, show distinct patterns of genomic changes, cancer-related signaling pathways, and clinical outcomes. This includes the identification of previously unknown, high-risk DZ-like cancers, as well as LZ-intermediate groups with better prognoses [31]. This framework provides a comprehensive understanding of how GC development relates to the emergence of B-cell lymphoma. This study also highlights how useful single-cell analyses are for improving molecular classification, refining prognostic predictions, and developing treatments tailored to the biological characteristics of DLBCL [31]. Importantly, while these models resolve tumor-intrinsic cellular states, they do not fully capture the complexity of the surrounding TME.

The comprehensive Ecotyper-analysis identified 44 unique cellular states across both cancerous and non-cancerous environments, which were then classified into nine consistent cellular ecosystems [32]. Each ecosystem demonstrated characteristic molecular subtypes, clinical outcomes, and treatment responses. These ecosystemic trends transcended conventional classifications based on cellular origin, illustrating that the biology of DLBCL is significantly influenced by interactions between neoplastic cells and their surrounding immune environment. These findings provide a crucial framework for incorporating cellular diversity into transcriptomic risk models (Figure 2) [32].

Despite these advances, each system of molecular classification describes different aspects of DLBCL biology and is associated with its own advantages and disadvantages, which may affect clinical application (Table 1). Genomic- and transcriptomic-based classification systems have considerably increased the biological understanding of DLBCL; however, there are several challenges to their widespread use in everyday clinical practice. LymphGen provides biologically meaningful genetic subtypes associated with pathogenic mechanisms and therapeutic vulnerabilities. However, its use requires detailed genomic profiling and sophisticated bioinformatic analysis, which may not be readily available in all diagnostic laboratories. Likewise, DHITsig improves the identification of high-risk germinal center-derived lymphomas compared with conventional cytogenetic double-hit classification, but is currently limited to specialized gene-expression platforms and requires further prospective validation in diverse patient populations.

Single-cell-based COO (sc-COO) models offer unprecedented resolution of lymphoma-cell states and developmental trajectories; however, single-cell transcriptomic approaches remain costly, technically demanding, and difficult to integrate into routine diagnostic workflows. Likewise, EcoTyper provides a unique ecosystem-level framework by incorporating tumor and microenvironmental cellular states, thereby capturing biological features not represented by tumor-intrinsic classifications alone. However, EcoTyper relies primarily on computational deconvolution of bulk transcriptomic data and requires additional validation in prospective clinical studies before broad implementation.

Importantly, these classification systems should be considered convergent rather than competing approaches. Genetic classifiers such as LymphGen define tumor-intrinsic oncogenic drivers; DHITsig identifies biologically aggressive germinal center-derived disease; sc-COO resolves cellular developmental states; and EcoTyper captures tumor ecosystem architecture and immune context. Future clinical classification frameworks will likely require integration of genomic, transcriptomic, single-cell, and spatial microenvironmental information to achieve robust biologic stratification and precision therapeutic selection in DLBCL.

Building on this ecosystem-based understanding of DLBCL, recent studies have further refined transcriptomic risk stratification at the clinical level. A recent investigation enhanced transcriptome-based risk stratification for newly diagnosed diffuse large B-cell lymphoma (ndDLBCL) by applying unsupervised clustering to extensive, well-characterized cohorts, leading to the identification of seven reproducible expression-defined categories [33]. Within this structure, the A7 cluster is recognized as a clinically aggressive, high-risk subtype, distinguished by an enrichment of activated B-cell-like characteristics, reduced immune infiltration, increased *MYC* expression, and recurrent copy-number alterations, features not fully encompassed by existing COO, International Prognostic Index (IPI), or genetic classification systems [33]. These findings highlight the need to integrate tumor-intrinsic programs with microenvironmental ecosystems, providing a foundation for spatially resolved profiling approaches that capture the spatial organization and functional interactions of tumor and immune cells in DLBCL.

### Spatial Regulation of B-Cell States in DLBCL

Spatial transcriptomics and multiplex imaging highlight the importance of the tissue microenvironment, showing that B-cell function is affected by its location in lymphoid tissues. GC zones, interfollicular regions, stromal niches, and vascular interfaces impose metabolic and signaling constraints that influence proliferation, selection, and differentiation [3]. Spatially resolved analyses show that immune and stromal environments are linked with malignant B-cell states and can promote immune evasion, treatment resistance, or aggressive growth, explaining why immune profiles are not always predictive [34,35]. These spatial technologies show that context-dependent states rather than fixed categories define B-cell identity. EcoTyper defines tumor ecosystems by deconvolving bulk transcriptomic data into distinct cell states and their higher-order organization, thereby capturing the cellular composition and microenvironmental context of DLBCL (Figure 2).

Building upon these transcriptome-defined and genetically resolved models of DLBCL heterogeneity, recent advances in multi-modal spatial profiling further extend this framework by integrating spatial organization and microenvironmental context, thereby linking molecular subtypes with tissue architecture and dynamic cell-cell interactions [26,34]. Recent advancements in multi-modal spatial profiling have provided new insights into the complex structure and function of the tumor immune microenvironment in DLBCL [34]. This has revealed a level of heterogeneity that goes beyond traditional classifications based on molecular characteristics and the cell type of origin [25,26,36]. Using highly multiplexed spatial transcriptomics, proteomics, and genomic analyses of a large group of tumors, Ref. [16] identified seven distinct cellular niches [16]. Unique cellular compositions, spatial distributions, and intercellular communication dynamics characterized each niche. These areas function as organized, multicellular ecosystems in which cancerous B cells, T lymphocytes, myeloid cells, and stromal components are arranged in a specific spatial pattern [16]. This spatial organization subsequently gives rise to niche-specific phenotypic states, which are influenced by both tumor-intrinsic programs and extrinsic microenvironmental factors [16]. Significantly, these results indicate that their transcriptional profiles or genetic predispositions do not exclusively dictate the functional attributes of malignant and immune-cell populations; rather, they are substantially influenced by their spatial positioning within the tumor’s architectural framework. In particular, tumors originating in immune-privileged locations displayed diffuse niches populated by activated and effector T cells coexisting with malignant B cells, thereby illustrating that immune cells can maintain a transcriptionally active yet spatially constrained state within defined microenvironmental contexts. These observations emphasize that immune regulation in DLBCL is governed by spatially organized patterns of cell-cell interactions, in which the arrangement of cellular neighborhoods governs processes such as activation, suppression, and intercellular communication within the tumor ecosystem (Figure 2) [15]. Furthermore, the delineation of these specific niches provides a crucial framework for elucidating how spatial heterogeneity shapes phenotypic diversity in both cancerous and non-cancerous cell populations. This underscores the notion that the evolution of DLBCL is governed by dynamic, context-sensitive interactions involving genetic modifications, transcriptional pathways, and the encompassing microenvironment. Overall, these insights highlight the critical role of spatial context in shaping DLBCL heterogeneity by integrating genetic alterations, transcriptional programs, and microenvironmental signals, thereby linking molecular programs to tissue architecture and cellular interactions.

## 3. Treatment Strategies for DLBCL

Treatment strategies for DLBCL have evolved substantially over the last two decades, with the emergence of immunotherapy, targeted drugs, antibody-based therapies, and cellular therapies. Even though rituximab-based chemoimmunotherapy remains the backbone of treatment for major newly diagnosed B-cell lymphomas, advances in molecular characterization have revealed the biologic heterogeneity of DLBCL, enabling the development of mechanism-based therapeutic approaches. Therefore, contemporary management integrates standard chemoimmunotherapy with targeted small molecule inhibitors, immune checkpoint modulation, antibody-based therapies, CAR T-cell therapy, and precision medicine strategies, with treatment selection increasingly guided by disease biology, patient fitness, and treatment setting.

### 3.1. Chemoimmunotherapy and Conventional Treatment Strategies

**Figure 3:**
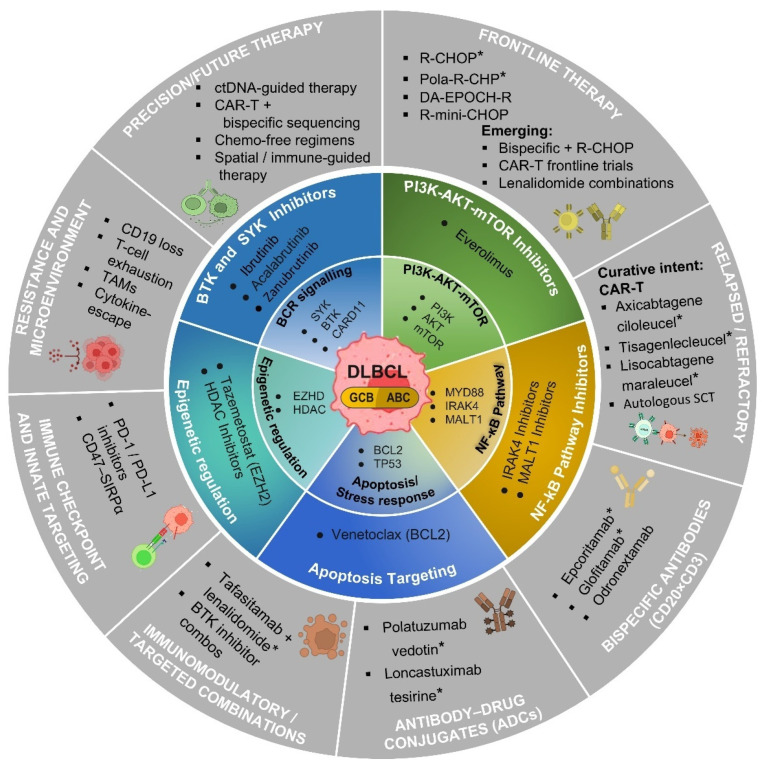
Current and emerging therapeutic landscape of diffuse large B-cell lymphoma (DLBCL). The central panel illustrates the key molecular and biological pathways involved in DLBCL pathogenesis, which include B-cell receptor (BCR) signaling, PI3K/AKT/mTOR signaling, NF-κB activation, apoptotic dysregulation, and epigenetic alterations. The middle ring highlights representative therapeutic targets and drug classes, while the outer ring defines current treatment strategies and emerging therapeutic approaches across frontline and relapsed/refractory settings. Therapies approved by the U.S. Food and Drug Administration (FDA) and/or the European Medicines Agency (EMA) for DLBCL are denoted by an asterisk (*). In contrast, drugs and strategies under clinical investigation are not. ADCs: antibody-drug conjugates; BCR: B-cell receptor; CAR-T: chimeric antigen receptor T-cell therapy; ctDNA: circulating tumor DNA; EZH2: enhancer of zeste homolog 2; HDAC: histone deacetylase; IRAK4: interleukin-1 receptor-associated kinase 4; mTOR: mammalian target of rapamycin; NF-κB: nuclear factor kappa B; PI3K: phosphoinositide 3-kinase; SCT: stem cell transplantation.

Frontline therapy for DLBCL (Figure 3) remains centered on rituximab-based chemoimmunotherapy, with the goal of cure in most patients [37,38]. R-CHOP remains a global standard and produces long-term survival in approximately 60–70% of patients in rituximab-era cohorts; however, outcomes vary significantly depending on clinical and biological risk. Survival is strongly influenced by the IPI, with 5-year overall survival ranging from approximately 80–90% in low-risk patients to approximately 25–50% in high-risk patients (IPI 4–5) [39,40]. Furthermore, adverse biological subgroups, particularly double-hit B-cell lymphoma and double-expressor lymphoma, are associated with significantly inferior outcomes compared to standard-risk DLBCL [41]. Dose-adjusted EPOCH-R (DA-EPOCH-R) has been evaluated as an intensified frontline regimen for DLBCL. However, in unselected DLBCL, the phase III CALGB/Alliance 50303 trial demonstrated no improvement in progression-free or overall survival compared to R-CHOP, with increased toxicity in the DA-EPOCH-R arm. Despite this, DA-EPOCH-R remains commonly utilized in high-risk subtypes such as primary mediastinal large B-cell lymphoma (PMBCL) and HIV-associated aggressive B-cell lymphomas, as well as HGBCL with *MYC*, *BCL2*, and/or *BCL6* rearrangements [42].

In limited-stage disease, treatment has shifted toward response-adapted strategies guided by interim positron emission tomography (PET). The FLYER trial [43] included patients aged 18–60 years with an age-adjusted IPI of 0 and non-bulky disease (<7.5 cm) and demonstrated that four cycles of R-CHOP followed by two doses of rituximab was non-inferior to six cycles of R-CHOP in this highly selected low-risk population, thereby supporting abbreviated therapy in this setting rather than its use as a general standard, whereas S1001 provides the key prospective PET-adapted evidence for a broader limited stage cohort in which radiotherapy can be omitted after a complete metabolic response. Accordingly, abbreviated treatment should not be generalized to all limited-stage DLBCL without attention to age, risk features, bulk, and PET response [44]. However, advanced-stage DLBCL is a biologically and clinically distinct entity in which response-adapted de-escalation is inappropriate, necessitating therapeutic intensification and the integration of novel agents.

In advanced-stage disease, antibody-drug conjugates have entered frontline treatment. POLARIX established polatuzumab vedotin plus R-CHP (pola-R-CHP) as a frontline option by improving progression-free survival over R-CHOP. However, the initial report did not show a statistically significant overall survival (OS) advantage [45], and this is consistent with the longer-term follow-up data presented in subsequent updates. Subgroup analyses (including ABC subtype, IPI 3–5, and age > 60 years) indicating differential benefit remain exploratory and should not be used alone to guide treatment selection outside of these populations [46]. Signals of preferential benefit in higher-risk groups, including ABC-type disease or double-expressor lymphoma, should therefore be described as exploratory rather than definitive. Biologically aggressive entities such as high-grade B-cell lymphoma (HGBCL) with *MYC* and *BCL2* and/or *BCL6* rearrangements have inferior outcomes with R-CHOP. They are often considered for intensified regimens, but evidence should be drawn from studies of those entities rather than from PMBCL (primary mediastinal large B-cell lymphoma) trials [47]. Disease biology and risk stratification not only influence the selection of systemic therapy but also play important roles in estimating the risk of central nervous system (CNS) relapse and in determining the need for CNS-directed prophylactic strategies in selected patients. Relapse in the CNS is a rare but clinically devastating complication of DLBCL, occurring in approximately 2–5% of patients and being associated with poor clinical outcomes. Risk stratification is commonly performed using the CNS International Prognostic Index (CNS-IPI), which integrates clinical and disease-specific risk factors to identify patients at increased risk of CNS relapse [48].

However, the optimal approach to CNS prophylaxis remains controversial. Although intrathecal methotrexate and high-dose intravenous methotrexate have historically been administered to high-risk patients, recent retrospective studies and contemporary analyses have questioned the magnitude of benefit associated with routine prophylactic use. Emerging data indicate that intrathecal or systemic methotrexate does not reliably decrease the risk of CNS relapse in all high-risk patient groups [49,50,51]. Thus, a growing number of clinicians are moving toward more selective, risk-adapted strategies for CNS prophylaxis, with treatment decisions guided by CNS-IPI risk assessment, high-risk extranodal sites, patient fitness, and overall disease biology. While CNS prophylaxis aims to prevent secondary CNS involvement in systemic DLBCL, primary CNS lymphoma (PCNSL) is a biologically and therapeutically distinct entity arising within the immune-privileged CNS microenvironment. Compared with systemic DLBCL, PCNSL is characterized by frequent MYD88 and CD79B mutations and often exhibits molecular features that overlap with the MCD genetic subtype [52]. Therapeutically, PCNSL differs fundamentally from systemic DLBCL and comprises high-dose methotrexate-based induction regimens, consolidation with autologous stem cell transplantation in selected cases, and novel targeted approaches, including BTK inhibitors (e.g., ibrutinib) and immunomodulatory agents (e.g., lenalidomide) [53]. These biological and therapeutic distinctions underscore the importance of considering PCNSL separately from systemic DLBCL when interpreting molecular studies and therapeutic outcomes. Besides disease stage and biological risk, patient-related factors such as age are also determinants of therapeutic decision-making in DLBCL.

In the treatment of older (>60 years) and frail patients, selecting therapies requires careful consideration of both their efficacy and patients’ ability to tolerate them. Treatment plans that adjust doses, such as R-mini-CHOP, have demonstrated feasibility and clinical activity, with clear benefits in this population [54]. In addition, emerging treatment strategies are exploring chemotherapy-sparing approaches by incorporating targeted agents and immunomodulatory therapies, including lenalidomide-based combinations [55]. For patients with relapsed or refractory (R/R) DLBCL, treatment selection depends on the timing of relapse, chemosensitivity, transplant eligibility, and access to cellular therapy. Salvage chemoimmunotherapy followed by autologous stem cell transplantation (ASCT) remains appropriate for selected patients with later relapse and chemotherapy-sensitive disease [56]. However, patients with primary refractory disease or relapse within 12 months after completion of first-line therapy should be evaluated for second-line CD19-directed CAR T-cell therapy when eligible. This approach is supported by product-specific randomized data, such as the ZUMA-7 and TRANSFORM trials, which demonstrated benefit for axicabtagene ciloleucel and lisocabtagene maraleucel, respectively. In contrast, the BELINDA trial did not show superiority of tisagenlecleucel over standard second-line therapy [57]. Patients who are not candidates for transplant or CAR T-cell therapy increasingly require individualized sequencing of antibody-drug conjugates, bispecific antibodies, immunomodulatory combinations, and clinical trials [41,58].

### 3.2. Targeted Small Molecule Therapies

Targeted therapies have become increasingly important in DLBCL, although their roles vary by biologic subgroup and line of therapy. In ABC-DLBCL, dysregulated BCR signaling, often driven by *CD79A/B* and *MYD88* alterations, promotes constitutive NF-κB activation and establishes a therapeutic vulnerability [59,60,61,62]. These observations provided the biological rationale for therapeutic targeting of Bruton tyrosine kinase (BTK), a central mediator of BCR signaling, leading to the clinical development of BTK inhibitors in DLBCL. The phase III PHOENIX trial investigated the combination of ibrutinib and R-CHOP in patients with previously untreated non-GCB DLBCL. Although the study did not demonstrate a survival benefit in the overall population, a prespecified subgroup analysis suggested improved event-free survival (EFS), progression-free survival (PFS), and overall survival (OS) among patients younger than 60 years receiving ibrutinib plus R-CHOP. In contrast, older patients experienced increased toxicity and reduced treatment intensity [63]. Despite these findings, BTK inhibition has not become a standard treatment across unselected DLBCL populations. More selective BTK inhibitors and combinations remain under investigation [64,65,66]. SYK, PI3K, and mTOR pathway inhibitors also provide biologically rational approaches. However, their clinical activity in aggressive lymphomas has been variable and should be presented as investigational or context-dependent rather than broadly established. The PI3K pathway activates the mammalian target of rapamycin (mTOR) pathway. The mTOR pathway subsequently regulates protein synthesis, cellular proliferation, and metabolic alterations within DLBCL. Although mTOR inhibitors such as temsirolimus and everolimus have shown limited effectiveness in treating relapsed/refractory DLBCL, research is ongoing into combination therapies. These therapies aim to improve treatment outcomes by targeting both the PI3K and mTOR pathways [60,67,68]. Further investigation into rational combination strategies is needed to understand their clinical efficacy.

Moreover, DLBCL is characterized by dysregulation of apoptotic control mechanisms, most prominently through aberrant *BCL2* expression. Venetoclax targets BCL2 and promotes mitochondrial apoptosis, but single-agent activity in DLBCL has been limited; current interest is greatest in rational combinations and biomarker-enriched settings [69,70,71]. Epigenetic alterations are also important, especially in GCB-like DLBCL, where EZH2 inhibition with tazemetostat has activity in selected patients [72,73]. Additional molecular targets, including IRAK4, MALT1, the NOTCH signaling pathway, and other targeted strategies, remain active areas of clinical investigation.

### 3.3. Innate and Adaptive Immune Checkpoint Targeting

New immunotherapeutic strategies are transforming DLBCL treatment by enhancing antitumor immunity and targeting the immunosuppressive mechanisms that shape the TME.

Immune checkpoint blockade is an important biologic strategy aimed at reversing tumor-induced immune suppression within the TME. In DLBCL, malignant B cells and associated immune cells can exploit inhibitory checkpoint pathways, particularly the PD-1/PD-L1 axis, to evade cytotoxic T-cell responses [74,75,76]. PD-1 blockade can reinvigorate T-cell function, but therapeutic efficacy differs substantially across lymphoma entities. Therefore, the biologic presence of a checkpoint pathway should be separated from proven clinical benefit in DLBCL, NOS.

PD-1 inhibitors have shown efficacy across several lymphoma subtypes; single-agent responses in DLBCL and NOS have generally been more modest than in classical Hodgkin lymphoma and PMBCL. Current developments in DLBCL are therefore focused on combinations with CAR T-cell therapy, bispecific antibodies, antibody-drug conjugates, and targeted agents rather than the routine use of PD-1 monotherapy. Other checkpoint molecules, including CTLA-4, LAG-3, and TIM-3, remain investigational in aggressive B-cell lymphomas. Their most plausible role may be in rational combinations designed to reverse deeper T-cell exhaustion, but clinical claims should remain cautious until supported by lymphoma-specific data [77,78].

PD-1-directed therapy primarily modulates adaptive immunity, whereas CD47-SIRPα blockade is intended to enhance innate immune clearance. CD47 is a ubiquitously expressed transmembrane protein that functions as a ‘don’t-eat-me’ signal, and DLBCL cells can overexpress CD47 to avoid macrophage-mediated elimination. Interaction between CD47 and signal regulatory protein alpha (SIRPα) transmits an inhibitory signal to macrophages and dendritic cells, preventing phagocytosis and enabling innate immune evasion [79,80,81,82].

Therapeutic approaches that block the CD47-SIRPα interaction, therefore, stop this inhibitory signaling, promoting macrophage recognition and the subsequent phagocytosis of cancer cells. After CD47 checkpoint inhibition, macrophages can respond to pro-phagocytic “eat-me” signals, such as calreticulin on the surface of tumor cells; this response then leads to antibody-dependent cellular phagocytosis and the destruction of tumor cells [83,84].

Magrolimab, a monoclonal antibody targeting CD47, has demonstrated proof of concept in blocking the CD47-SIRPα innate immune checkpoint, particularly when combined with rituximab. Early clinical studies demonstrated the feasibility of enhancing macrophage-mediated phagocytosis through this pathway. However, its further development in diffuse large B-cell lymphoma has been limited by a lack of confirmatory efficacy in later-stage studies and the discontinuation of its DLBCL development program, indicating that it should not be considered an established therapeutic option in this setting. The clinical translation of CD47-SIRPα blockade in DLBCL remains uncertain. Nevertheless, the biological rationale is supported by preclinical evidence showing enhanced macrophage phagocytosis and potential synergy with tumor-opsonizing antibodies, and the pathway continues to be explored through next-generation anti-CD47 and anti-SIRPα agents in early-phase clinical development [85,86,87].

DLBCL-TME frequently harbors immunosuppressive macrophages and T cells exhibiting exhaustion, which collectively facilitate immune evasion. Consequently, targeting of both adaptive and innate immune checkpoints, including PD-1 and the CD47-SIRPα axis, could potentially circumvent these suppressive mechanisms, thereby enhancing therapeutic efficacy, especially in the context of relapsed/refractory disease [85,88].

### 3.4. Antibody-Drug Conjugates and Bispecific Antibodies

#### 3.4.1. Bispecific Antibodies

An integrative genomic and transcriptomic analysis of DLBCL establishes a biologically and clinically significant classification framework based on immune contexture, referred to as “immune quadrants” (IQs), which amalgamate COO (ABC vs. GCB) with immune activity (hot vs. cold) (Figure 3). RNA sequencing studies that examined multiple patient groups have identified four distinct subgroups. Specific genetic changes and features of the tumor environment define these subgroups. This highlights how changes within the tumor itself affect the immune system’s characteristics. For example, *SOCS1* mutations in immune-active GCB tumors and MYC-driven programs in immune-cold disease are key factors. This perspective provides crucial clinical insight, demonstrating that tumors with prior immune activation are more likely to respond to immunotherapeutic interventions [14].

Consequently, CD20xCD3 bispecific antibodies provide a rational off-the-shelf strategy by redirecting endogenous T cells toward malignant B cells [89]. Glofitamab and epcoritamab have demonstrated clinically meaningful response rates and currently have the clearest U.S. regulatory footing in DLBCL after at least two prior systemic lines. In contrast, mosunetuzumab is approved for relapsed/refractory follicular lymphoma, whereas odronextamab has shown encouraging activity in early-phase studies. However, its regulatory status in DLBCL requires interpretation based on indication and jurisdiction, as odronextamab received conditional EMA approval in 2024 for relapsed/refractory DLBCL after at least two prior systemic therapies, while the FDA issued a Complete Response Letter requesting additional data. These agents are generally associated with manageable toxicity profiles, particularly cytokine release syndrome, which can be mitigated through step-up dosing and appropriate monitoring strategies. The bispecific antibody landscape now also includes randomized evidence: in the STARGLO trial, the addition of glofitamab to gemcitabine-oxaliplatin (GemOx) improved overall survival, progression-free survival, and complete response rate compared with R-GemOx in transplant-ineligible relapsed/refractory DLBCL, moving glofitamab beyond promising single-arm data [90].

#### 3.4.2. Antibody-Drug Conjugates

Antibody-drug conjugates (ADCs) deliver cytotoxic payloads directly to malignant B cells and can remain useful when T-cell-directed approaches are not feasible or have failed. Polatuzumab vedotin targets CD79b, and loncastuximab tesirine targets CD19, enabling selective intracellular delivery of cytotoxic agents [14,58,91]. ECHELON-3 further expanded non-CAR-T options for multiply relapsed large B-cell lymphoma by demonstrating that brentuximab vedotin plus lenalidomide and rituximab improved overall survival compared with lenalidomide-rituximab alone in patients ineligible for ASCT or CAR T-cell therapy [92]. ADCs should therefore be discussed as product-specific agents with distinct targets, data, and clinical roles rather than as an interchangeable class.

### 3.5. CAR-T Therapy

Chimeric antigen receptor (CAR) T-cell therapy (Figure 3) has become a significant advancement in the treatment of aggressive B-cell lymphomas, particularly DLBCL. This methodology employs autologous T lymphocytes genetically modified ex vivo to express chimeric receptors targeting tumor-associated antigens, predominantly CD19, which are then reinfused following lymphodepleting chemotherapy. In contrast to conventional therapies, CAR T cells identify malignant cells in a major histocompatibility complex (MHC) independent manner, allowing for direct and highly specific tumor cell targeting [93,94,95].

Initial studies such as ZUMA-1, JULIET, and TRANSCEND NHL 001 established the activity of CD19-directed CAR T-cell therapy in relapsed/refractory large B-cell lymphoma [94,96,97]. In the second-line setting, however, evidence is product-specific rather than class-uniform. ZUMA-7 showed that axicabtagene ciloleucel (axi-cel) improved event-free survival and durable overall survival compared with standard second-line chemoimmunotherapy followed by ASCT in patients with primary refractory or early-relapsed large B-cell lymphoma [57]. Long-term follow-up of ZUMA-7, conducted at a median follow-up of 47.2 months, demonstrated a prolonged overall survival benefit, with a 4-year overall survival rate of 54.6% for axi-cel compared with 46% for standard therapy, representing an absolute improvement of 8.6 percentage points (HR 0.73, 95% CI 0.54–0.98) [98]. Similarly, the 3-year follow-up of TRANSFORM showed durable efficacy with lisocabtagene maraleucel (liso-cel), particularly with respect to EFS and PFS. The 36-month PFS rate was 51% with liso-cel compared with 26.5% with standard therapy. Although overall survival numerically favored liso-cel (36 months overall survival rate, 63% vs. 52%), median overall survival was not reached in either treatment arm, and the difference was not statistically significant (HR, 0.757; 95% CI, 0.481–1.191). The interpretation of the overall survival results is further complicated by substantial crossover, with 66% of patients in the standard-of-care arm subsequently receiving liso-cel. In contrast, BELINDA did not show superiority of tisagenlecleucel over standard therapy [99,100]. However, when interpreting BELINDA, important design differences should be considered, such as the increased use of bridging therapy and the longer time from leukapheresis to CAR T-cell infusion compared to ZUMA-7 and TRANSFORM, which may have contributed to the negative outcome. Modern treatment sequencing should therefore distinguish axi-cel and liso-cel as positive randomized second-line products rather than assuming a uniform class effect across all CD19-directed CAR T-cell therapies.

Despite these advancements, the wider use of CAR T-cell therapy faces several challenges. Cytokine release syndrome (CRS) and immune effector cell-associated neurotoxicity syndrome (ICANS), both negative effects of the treatment, require specialized management plans and infrastructure [101]. Consequently, the complex and individualized nature of the manufacturing process introduces logistical challenges and delays that may limit its universal application. Moreover, the lasting effectiveness of treatments is also limited by biological resistance mechanisms. These changes include the loss of antigens, such as the decrease in CD19, T-cell exhaustion, and the development of an immunosuppressive TME signature [95,102]. These constraints have prompted research into next-generation cellular therapies and rational combination approaches. A summary of the landmark clinical trials that have established current standards of care and emerging therapeutic strategies in DLBCL is presented in Table 2.

### 3.6. Precision Medicine and Future Directions

Despite the incorporation of rituximab into frontline therapy, approximately 30% of patients with DLBCL relapse or develop refractory disease following initial treatment [105]. However, this overall estimate masks substantial heterogeneity driven by both clinical and biological factors. Patients with low-risk disease, particularly GCB DLBCL, and low IPI scores have favorable outcomes, with fewer treatment failures. In contrast, patients with ABC/non-GCB DLBCL and high-risk clinical features have significantly higher rates of relapse or refractory disease. This variability reflects the underlying molecular complexity of DLBCL and highlights the need for risk-adapted therapeutic strategies [41]. Genomic profiling has identified pathways driving progression and resistance, including BCR/NF-κB signaling, PI3K/AKT/mTOR signaling, apoptosis control, epigenetic regulation, and immune escape [106]. DLBCL is therefore best understood as a diagnostic umbrella containing multiple biologically distinct diseases, making molecular stratification central to modern management rather than a purely descriptive exercise. The practical implication is that pathology is no longer limited to naming the disease; it now includes a complete work-up that connects morphology, immunophenotyping (IHC), COO assignment, key genomic alterations, and TME context, and ensures appropriate tissue triage for molecular testing, because these readouts increasingly forecast prognosis, identify vulnerabilities (e.g., BCR/NF-κB/JAK-STAT signaling, apoptosis control, immune escape), and set the stage for rational treatment refinement, especially when relapse occurs [107]. This molecular and microenvironmental view aligns directly with the current immunotherapy era: response is not only about the target (CD19/CD20) but also about the tumor’s biology and the immune ecosystem in which the therapy must function.

Innate immune strategies make sequencing and combination design as much a biology-driven problem as a drug availability problem. Precision medicine is increasingly shaping DLBCL treatment, with therapeutic choices informed by molecular classification, tumor-microenvironment features, and dynamic response assessment. Genomic and transcriptomic profiling have refined subclassification beyond conventional COO categories, identifying biologically distinct subgroups with different vulnerabilities [21,25]. ctDNA-based measurable residual disease (MRD) is rapidly emerging as a clinically actionable response biomarker in large B-cell lymphoma, with the integration of ctDNA into response-adaptive strategies. ctDNA-based MRD is emerging as a promising biomarker for disease monitoring, risk stratification, and early relapse detection in DLBCL. Recent prospective studies have demonstrated the strong prognostic value of ctDNA dynamics following frontline therapy. However, despite these encouraging findings, the clinical utility of ctDNA-guided treatment adaptation remains investigational, as no prospective randomized trial has yet demonstrated improved patient outcomes with ctDNA-directed therapeutic intervention. Several ongoing prospective studies are evaluating whether response-adapted treatment strategies guided by ctDNA kinetics can improve clinical outcomes and further define the role of ctDNA-MRD in precision management of DLBCL [108,109].

Single-cell and spatial profiling have transformed our understanding of tumor heterogeneity and immune interactions by showing that therapeutic response depends on both tumor-intrinsic programs and the immune ecosystem in which treatment occurs. Emerging data also underscore the need for deeper biologic stratification: single-cell analyses in primary refractory DLBCL link *C1QB*-positive macrophage programs and lipid metabolism-driven immunosuppression to resistance to immunotherapeutic approaches, highlighting potentially actionable microenvironmental mechanisms [110]. Rational combination therapy is also advancing beyond conventional salvage frameworks. In the phase Ib ViPOR study (*n* = 50), conducted in heavily pretreated patients with relapsed/refractory B-cell lymphomas, the combination of venetoclax, ibrutinib, prednisone, obinutuzumab, and lenalidomide demonstrated encouraging activity. Notably, higher complete response rates were observed in patients with non-GCB DLBCL and *MYC/BCL2-*rearranged HGBCL; however, these findings are based on small subgroup sizes and require validation in larger prospective studies [111]. As the therapeutic landscape evolves, the most significant remaining challenge is biology-driven optimization of treatment sequencing and rational combination design, which remains an area of active investigation rather than a fully established paradigm. In this context, integrating molecular classification, immune profiling, ctDNA monitoring, and TME characterization will be increasingly important for optimizing patient selection, therapeutic sequencing, and combination strategies. Furthermore, larger and more diverse cohorts, including Asian populations, will be essential for validating emerging pathogenomic mechanisms and optimizing therapy across clinical settings.

## 4. Conclusions

DLBCL is not a single disease but a dynamic, ecosystem-driven malignancy in which the integration of tumor genetics and the TME dictates clinical behavior and therapeutic response. Rationally designed combination strategies that simultaneously target tumor cell-intrinsic vulnerabilities and microenvironmental resistance mechanisms will be essential to overcome therapeutic failure. In this context, DLBCL serves as a paradigm for precision immuno-oncology, where the convergence of genomic architecture, immune ecology, and spatial biology will define the next generation of personalized therapeutic interventions and ultimately improve patient outcomes.

## Figures and Tables

**Table 1:** Comparison of major biological and molecular classification frameworks in DLBCL.

Classification Framework	Key Strengths	Major Limitations	Clinical Relevance	Technology Requirements	Current Clinical Utility
Cell-of-Origin (COO) (GCB/ABC) [18]	Widely adopted; clinically accessible; established prognostic value; adaptable to IHC-based algorithms	Limited biological resolution; does not capture genetic heterogeneity or tumor microenvironmental complexity	Baseline risk stratification and subtype assignment	Gene expression profiling (GEP)	Established clinical standard
LymphGen [26]	Links recurrent mutations to pathogenic mechanisms, therapeutic vulnerabilities, and clinical outcomes; improves biologic classification beyond COO	Requires comprehensive genomic profiling and advanced bioinformatic infrastructure; limited availability in routine diagnostics	Molecular risk stratification and precision therapeutic selection	Targeted NGS, WES/WGS, bioinformatic analysis	Advanced translational application in the genetic classification of B-cell lymphoma
DHITsig [29]	Identifies biologically aggressive germinal center-derived lymphomas beyond conventional *MYC/BCL2* FISH-defined double-hit disease	Dependent on specialized transcriptomic platforms, it requires broader prospective validation	Prognostic refinement and identification of high-risk disease	GEP/n-Counter	Moderately validated
Single-cell COO (sc-COO) [31]	Provides unprecedented resolution of lymphoma cell states	Expensive, technically demanding, and currently difficult to implement in routine clinical workflows	Biological discovery and future precision classification	Single-cell RNA sequencing and computational analysis	Emerging research framework
EcoTyper [32]	Integrates malignant, immune, and stromal cell states; captures ecosystem-level interactions associated with outcome and therapeutic response	Relies on computational deconvolution of bulk transcriptomic data; requires further prospective clinical validation	Microenvironment-guided risk stratification and immunotherapy development	Bulk RNA sequencing with computational ecosystem deconvolution	Emerging translational framework

**Table 2:** Landmark clinical trials and the current therapeutic roles of established and emerging treatments in DLBCL.

Therapy	Clinical Trial	Line of Therapy	Comparator	Critical Outcomes	Clinical Role	Reference
R-CHOP	LNH98-5	Frontline DLBCL	CHOP	The addition of rituximab significantly improved overall survival and event-free survival	Global frontline standard	[37]
Polatuzumab vedotin + R-CHP (pola-R-CHP)	POLARIX	Frontline advanced-stage DLBCL	R-CHOP	Improved progression-free survival compared with R-CHOP	Frontline option, especially in higher-risk disease	[45]
Axicabtagene ciloleucel (axi-cel)	ZUMA-7	Second-line early relapsed/refractory (R/R) DLBCL	Salvage chemotherapy + ASCT	Superior event-free survival and durable overall survival	Preferred second-line CAR T-cell therapy	[57]
Lisocabtagene maraleucel (liso-cel)	TRANSFORM	Second-line early R/R DLBCL	Standard salvage therapy + ASCT	Improved event-free survival, complete response rate, progression-free survival and numerically improved overall survival	Preferred second-line CAR T-cell therapy	[103]
Tisagenlecleucel (tisa-cel)	BELINDA	Second-line early R/R DLBCL	Standard salvage therapy + ASCT	Did not demonstrate superiority over standard therapy	Limited second-line role	[99]
Glofitamab + GemOx	STARGLO	Transplant-ineligible R/R DLBCL	R-GemOx	Improved overall survival, progression-free survival, and complete response rate	Later-line bispecific antibody option	[90]
Brentuximab vedotin + Lenalidomide + Rituximab	ECHELON-3	Multiply relapsed large B-cell lymphoma (LBCL)	Lenalidomide + Rituximab	Improved overall survival	Non-CAR-T option in later lines	[92]
Loncastuximab tesirine	LOTIS-2	Relapsed/Refractory DLBCL	Single-arm study	Durable responses in heavily pretreated patients	Later-line antibody-drug conjugate (ADC)	[91]
Tafasitamab + Lenalidomide	L-MIND	Transplant-ineligible R/R DLBCL	Single-arm study	Durable responses and prolonged survival	Later-line immunotherapy option	[104]

## Data Availability

No new data were generated or analyzed in this study.
